# Sensory and emotional responses to deep pressure stimulation at myofascial trigger points: a pilot study

**DOI:** 10.3389/fnins.2023.1197302

**Published:** 2023-07-07

**Authors:** Seoyoung Lee, Heeyoung Moon, Yeonhee Ryu, In-Seon Lee, Younbyoung Chae

**Affiliations:** ^1^Acupuncture and Meridian Science Research Center, Kyung Hee University, Seoul, Republic of Korea; ^2^KM Science Research Division, Korea Institute of Oriental Medicine, Daejeon, Republic of Korea

**Keywords:** deep pressure, pain, pleasantness, trigger point, muscle properties

## Abstract

**Objective:**

Although manual pressure, such as that used during a massage, is often associated with pain, it can simultaneously be perceived as pleasant when applied to certain body areas. We hypothesized that stimulation of myofascial trigger points (TPs) leads to simultaneous pain and pleasure. TPs are hyperirritable points located in the taut band of the skeletal muscle.

**Method:**

In this study, we measured the muscle tone, muscle stiffness, and pressure pain threshold of TPs and control points in the left brachioradialis muscle of 48 healthy participants. We also applied deep compression to the two points and collected subjective data on pain, pleasantness, unpleasantness, and relief.

**Result:**

Greater muscle stiffness was observed in the TPs versus control points (*t* = 6.55, *p* < 0.001), and the pain threshold was significantly lower in the TPs (*t* = −6.21, *p* < 0.001). Unpleasantness ratings after deep compression were significantly lower in the TPs compared with control points (*t* = −2.68, *p* < 0.05). Participants experienced greater relief at the TPs compared with control points (*t* = 2.01, *p* < 0.05), although the perceived pain did not differ between the two types of points.

**Conclusion:**

We compared the properties of TPs and control points, and found that deep compression at TPs was associated with higher muscle tone and stiffness, lower unpleasantness ratings, and higher relief ratings compared with the control points. These findings suggest that, at least for some TPs, pain and pleasantness are simultaneously elicited by deep pressure stimulation.

## 1. Introduction

Pain and pleasantness are generally considered incompatible states. The International Association for the Study of Pain defined pain as “an unpleasant sensory and emotional experience associated with, or resembling that associated with, actual or potential tissue damage” (Raja et al., 2020). That is to say, pain generally signifies unpleasantness, so the concept of “pleasant pain,” i.e., the pleasantness of a painful experience, may appear to be a paradox. However, a previous study demonstrated that pain could be pleasant when elicited in the context of relative relief (Leknes et al., 2013). Another study involving participants who were aware that acupuncture can have therapeutic effects showed that some participants experienced the sensation of acupuncture as pleasant (Takakura et al., 2013). In addition, even when pain is part of the experience, people tend to perceive manual pressure, such as massage, as simultaneously pleasant when applied to specific body areas (Mackenzie, 2009).

Deep pressure is involved in various forms of social touch, such as hugging and handholding, and can be applied in manual treatments such as massage. Both massage and social touch have been shown to reduce stress and pain (Suresh et al., 2008; Field, 2019). Previous researches have reported that moderate pressure massage is more effective than light pressure massage for decreasing anxiety, heart rate, and stretch reflex, as well as the electroencephalogram relaxation response (Lidbeck, 2002; Diego et al., 2004; Field, 2014). Furthermore, a study with healthy subjects showed that participants reported pleasure during deep compression (Case et al., 2021). Specifically, brain activation during deep compression was similar to that during C-tactile touch (a tactile experience hypothesized to convey pleasant emotions), indicating that the affective aspects of deep pressure may be reliant on the social context (Pawling et al., 2017; Case et al., 2021). During a back massage, amygdala and basal forebrain blood flow changes were associated with a reduction in heart rate (Ouchi et al., 2006).

Myofascial trigger points (TPs) are hyperirritable spots usually found in the taut band of skeletal muscle or fascia, that can cause referred pain through compression (Lavelle et al., 2007). Previous studies have reported lower pressure pain thresholds (PPTs) and higher muscle stiffness in TPs compared with adjacent muscle areas (Hong, 1998; Grabowski et al., 2018). Manual therapies such as ischemic compression and massage can be effective for treating TPs (Fernández-de-las-Peñas et al., 2005). Ischemic compression is administered by increasing deep pressure on the TPs until the maximum tolerable level of pain has been reached (Cagnie et al., 2013). Ischemic compression increased the PPT, range of motion, and blood flow at the TP, and reduced subjective pain (Fernández-de-las-Peñas et al., 2006; Moraska et al., 2013; Cagnie et al., 2015; Moraska et al., 2017). Although various studies have characterized and examined the treatment of TPs, the emotional responses elicited by the application of deep pressure on TPs have not been described.

The aim of this study was to determine whether painful deep pressure was more pleasurable at TPs compared with control points. We hypothesized that TPs would be related to pleasant pain, as these are the areas where people often voluntarily seek pressure application, even though the experience is painful. Thus, we compared TPs and control points in terms of muscle tone, muscle stiffness, and PPTs. To test our hypotheses, we measured subjective ratings of pleasantness, unpleasantness, and relief after manual deep pressure in both TPs and control points.

## 2. Methods

### 2.1. Participants

Forty-eight healthy right-handed participants (26 females) were recruited through an online advertisement entitled “A study investigating the tactile/emotional/physical properties of trigger points.” In order to control for potential differences in muscle properties and brain lateralization related to hand dominance, we included right-handed participants for this study and a follow-up functional magnetic resonance imaging (fMRI) study. The exclusion critieria were as follows: body mass index (BMI) < 18.5 or > 25 kg/m^2^; psychological or psychiatric disorders, vascular disorders, epilepsy, dementia, substance dependence, previous history of brain surgery; cognitive impairment; skin pathologies or sensory abnormalities; and doctors of Korean Medicine or students majoring in Korean Medicine (to exclude participants who had formal knowledge of acupuncture points and TPs). All participants provided written informed consent prior to the study, which was conducted in accordance with guidelines issued by the Human Subjects Committee and approved by the Institutional Review Board of Kyung Hee University, Seoul, Republic of Korea (approval number: KHSIRB-21-243).

As this research is a pilot study, we did not have reference to estimate the effect size. We estimated 48 participants by sample size calculations through power analysis with power level of 90% to detect an estimated effect size of *d* = 0.5, also accounting for a 10% of data loss.

### 2.2. Experimental design

**Figure 1:**
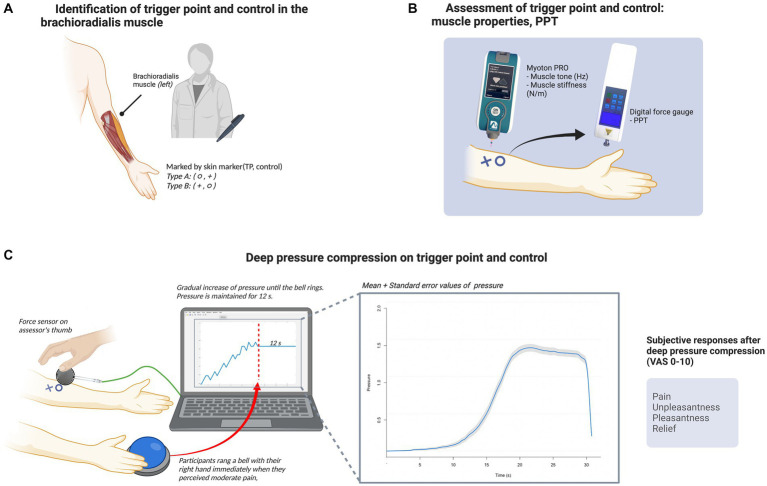
Experimental procedure. **(A)** Identification of the TP and control point in the brachioradialis muscle. A doctor of Korean Medicine detected the TP and control point in the left brachioradialis muscle of each participant, and marked the points with a circle or cross according to the subject group. **(B)** Assessment of the TP and control point: muscle properties and pressure pain threshold. Subsequently, an independent assessor measured muscle tone and stiffness in the two areas using the Myoton PRO device (Myoton AS) and assessed the pressure pain threshold using a digital force gage. **(C)** Deep compression at the TP and control point. Deep compression was applied to the two areas via the assessor’s right thumb. A force sensor was placed between the participant’s skin and the assessor’s thumb to monitor real-time pressure data using the UNO device (Arduino) and Matlab R2020a (MathWorks). Compression was gradually increased until the participant perceived moderate pain, at which point they immediately rang a bell with their right hand; the assessor then stopped increasing the pressure and maintained it for 12 s. The line graph *(left)* inside the monitor is an example of pressure monitoring during the trial, and the other line graph *(right)* shows the mean pressure values (blue line) and with standard error (gray shaded area). Following this procedure, the participants were asked to provide subjective ratings of pain, unpleasantness, pleasantness, and relief for each form of deep compression. The figure was created with BioRender (www.biorender.com).

This randomized, double-blind, crossover study was designed to compare psychophysical responses after deep pressure between TPs and control points in the left brachioradialis muscle. The study was conducted from August 2021 through September 2021. The study took place over 1 day for each participant and was conducted at the Korean Medicine Department of Kyung Hee University. On arrival, the participants received an explanation of the study procedure. After enrollment, we used an Excel random number generator to randomly assign each participant to either the type A (TP first) or type B (control point first) group. This was done to counterbalance the order of the two conditions. The study was conducted in the following order: questionnaire and baseline measures; identification of TP and control point; analysis of muscle properties; measurement of PPT; deep compression; and subjective ratings of deep compression. The participants were asked to sit on a chair with their left elbow flexed at nearly 90°, the lateral side of their left forearm facing up, and the medial side of their forearm gently resting on a table. Each participant maintained this position with their arm relaxed throughout the study. The experimental procedure is depicted in Figure 1.

We have selected the brachioradialis muscle as the target muscle among the many muscles in the body that have latent TPs. First of all, we did not want to incite unfavorable emotions in the participant through physical contact from the experimenter, who will be a stranger to them, as the emotional responses are crucial reports in this study. A quantified map illustrating where social touch is permitted in each body region depending on the relationship was published in a previous cross-country study. This study suggests that an emotionally distant person tends to only touch you in the arm and hand regions, so we looked for a muscle that corresponds to these regions (Suvilehto et al., 2015). In particular, the brachioradialis muscle was chosen because it can exert deep pressure in experimental settings and during fMRI scanning. Additionally, both healthy participants and participants with lateral epicondylitis were found to have a high prevalence of trigger points in the brachioradialis muscle. According to Aggarwal et al., among the brachioradialis, triceps brachii, supinator, and extensor carpi radialis brevis muscles in patients with Lateral Epicondylitis, the brachioradialis muscle was the area with the highest prevalence of trigger points (Aggarwal et al., 2020). All of the recruited adult participants also had a latent trigger point in the brachioradialis muscle, according to another study on healthy subjects. As a result of its advantages in terms of location and high prevalence, we decided to use the brachioradialis muscle (Kao et al., 2007).

### 2.3. Questionnaire and baseline measures

The participants completed the Social Touch Questionnaire (STQ) (Vieira et al., 2016). To measure subjective pain in the forearm, we asked the participants to rate their pain using a visual analog scale (VAS; 0–10 cm). Additionally, the participants were asked whether they had previously experienced a massage, and how they felt about it if yes (VAS; −5 to +5 cm; −5 = most negative and + 5 = most positive). We measured the percentage of fat and muscle quality in the under-arm area of each participant’s left forearm using the SKULPT device (Skulpt, San Francisco, CA, United States).

### 2.4. Identification of trigger and control points

A researcher with a Korean Medicine doctor’s license and over 5 years of clinical experience (H.M.) identified the TPs and control points in the left brachioradialis muscle. The TPs were identified based on criteria for discriminating latent myofascial TPs used in a previous study (Albin et al., 2020). TPs were selected in areas with a taut band and hypersensitive spot. Several potential TPs were identified via palpation and then compressed.

Each participant was asked to express their pain verbally during compression to assess the hypersensitivity of the points. Subsequently, control points were selected in areas without a taut band, and with no hypersensitivity, located 2 cm from TP points. In most cases, the TP was close to acupoint LI10 and the control point was medial to the TP.

The control points were determined in an area within 2 cm of the TP. We wanted to explore the distinct characteristics of TP in terms of muscle properties and emotional response during deep pressure compression. We decided to locate the control point in an adjacent area within the same muscle, so that we can control other differences between the two points. The within 2 cm criteria was empirically set within researchers as we agreed that it was enough distance within the same muscle and distance from the TP and also we followed similar previous studies criteria uses 1–2 cm distance from the TP to find the control (Ibarra et al., 2011). We demonstrated the spatial patterns of muscle stiffness over the region of interest over brachioradialis muscle (Supplementary Figures).

The assessor was kept in a separate room until the identification of the TP and control was complete in order to achieve blind assessment. In order to prevent the assessor from recognizing the points, the identified points were additionally marked with a circle (○) or cross (+) using a skin marker (TP and control point, respectively, for type A participants, and vice versa for the type B participants), so that the points cannot be recognized by the assessor. We standardized the protocol so that every assessment was carried out in the same order, with the circle always being completed before the cross.

### 2.5. Deep compression on trigger and control points

All participants received deep compression on both areas (the area marked with a circle first followed by the area marked with a cross). Deep pressure was applied through the assessor’s thumb until the participant perceived moderate pain. The participant immediately rang a bell with their right hand when they perceived moderate pain, which signaled to the assessor to stop increasing pressure and maintain the same level thereof for 12 s. To confirm an even increase in pressure and maintain the pressure corresponding to a moderate pain level for 12 s, we monitored the pressure using a digital pressure sensor consisting of the UNO device (Arduino®, New York, NY, United States), a force-sensitive resistor (FSR402; Interlink Electronics, Camarillo, CA, United States), and Simulink software (MathWorks, Natick, MA, United States). The thin sensor of the force-sensitive resistor was placed directly under the assessor’s thumb. During compression, pressure was delivered to the sensor, which decreased the resistance. To measure the force, the resistance was calculated as an output voltage.

Deep compression was applied to both areas -first to the area with a circle, then to the area with a cross-on all participants. For both TP and control point, we applied deep pressure until the participant felt moderate pain in order to achieve our goal to create a similar level of pain perception.

### 2.6. Assessment of muscle properties and pressure pain threshold

After the points had been marked, another researcher (S.L.) evaluated the physical properties of the TP and control point. The assessor measured the muscle quality in each area using the Myoton PRO device (Myoton AS, Tallinn, Estonia), starting with the circle marker, which indicated the TP for group A and control point for group B. When the thin probe of the Myoton PRO was positioned over each area perpendicularly with slight compression (0.18 N), the device delivered a weak force (0.4 N) for 15 ms, which damped natural oscillation in the underlying tissue. The device software measures the tissue response and calculates the viscoelastic properties (Schneider et al., 2015). As the assessor conducted the measurement, the researcher who identified the TP recorded the average muscle tone, stiffness, elasticity, relaxation, and creep values shown on the screen of the Myoton PRO.

We recorded muscle tone and muscle stiffness of all participants. Muscle tone (Hz) is characterized as the intrinsic state of resting tension without voluntary contraction and represents the natural oscillation frequency (Bailey et al., 2013). The muscle’s resistance to an applied deforming force is measured by its stiffness (N/m; Agyapong-Badu et al., 2013). We recorded both indices to measure both tension and tissue resistance of TP and control since multiple studies using myotonography on TP have shown increased muscle tone and stiffness compared to non-TPs.

To assess the pain sensitivity at each point, a digital force gage (Yueqing ALIYIQI Instrument Co., Zhejiang, China) was used to measure the PPT of the TP and control point. The assessor placed the probe of the device on each point on the left forearm and increased the pressure constantly until the participant initially felt a moderate level of pain (numeric rating scale score of 4 out of 10) and rang the bell positioned by their right hand. The final pressure was documented by the researcher, and the participant was asked to rate the pain they felt during the measurement using a VAS (0–10 cm). The participants were asked to remember the sensation felt during this procedure and consider it to correspond to ‘moderate’ pain during the rest of the study.

### 2.7. Psychophysical measurements after deep compression

To measure the psychophysical response to deep compression, we asked the participants to fill out a questionnaire pertaining to their subjective responses immediately after each compression trial. They reported how they felt during the deep compression of each TP and control point. The subjective responses included pain, pleasantness, unpleasantness, and relief of pain. The responses were recorded using a VAS (0–10 cm; 0 = none and 10 = the highest possible amount).

### 2.8. Preference and blinding of the TP and control point

After the participants had provided their subjective responses, they were asked to choose “which point (TP or control point) produced a sensation that was preferable during deep compression.” Additionally, we assessed whether participant blinding was achieved via a brief interview. At the end of the task, we asked the participants if they knew “which point was the TP and which point was the control.” If they said no, we asked them to guess which marked area was the TP.

### 2.9. Statistical analysis

To compare the physical properties and responses to deep compression between the TP and control point, we used a paired *t*-test for the muscle tone and stiffness values, PPT, and subjective responses (as measured by the VAS). A paired *t*-test was also performed to examine the order effect. Here, we report the 95% confidence intervals (CIs) for the differences in means. The effect size was measured using Cohen’s d, with |*d*| < 0.2 considered negligible, |*d*| < 0.5 considered small, |*d*| < 0.8 considered moderate, and all other values considered large (Cohen, 1992). To determine whether participant preferences significantly differed from the chance level, we applied the chi-square goodness of fit test. Preprocessing of the Arduino pressure values was conducted using Matlab R2020a software (MathWorks). For the statistical analyses, we used the rstatix and ggpubr packages in R (version 4.0.3; https://www.R-project.org/). Unless stated otherwise, all values are reported as means ± standard errors. *p*-values <0.05 were considered statistically significant.

## 3. Results

### 3.1. Baseline characteristics

The baseline characteristics of the participants are presented in Table 1. Paired *t*-tests were applied to test for order effects and analyze all subjective ratings (pain, unpleasantness, pleasantness, relief). We found no significant differences between group A (TP first) and group B (control point first) (*p* > 0.05).

**Table 1:** Characteristics of participants.

Characteristics		Number (%) or mean value ± standard deviation
Sex	Female	26 (54.17%)
	Male	22 (45.83%)
Age (years)		22.52 ± 2.34
Body Mass Index		21.37 ± 1.61
Percent body fat (lower arm)		18.82 ± 5.51
Arm pain (VAS, 0–100)		0.95 ± 3.81
Social Touch Questionnaire (0–80)		37.3 ± 8.61
Massage Experienced		46 (95.83%)
Massage Preference (VAS, −5 cm - +5 cm)		25.32 ± 24.21

### 3.2. Properties of TPs and control points

**Figure 2:**
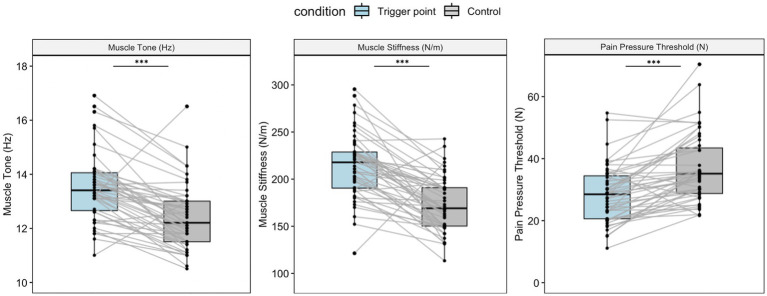
Muscle property measurements for the TP and control point. Boxplots show the median and interquartile range. Both muscle tone and stiffness were significantly higher at the TP compared with the control point (*t* = 6.55, *p* < 0.001, 95% CI: 0.8 to 1.51; *t* = 7.32, *p* < 0.001, 95% CI: 31.57 to 55.51). The pressure pain threshold was higher at the control point compared with the TP (*t* = −6.21, *p* < 0.001, 95% CI: −11.27 to −5.75). All *p*-values were acquired via pairwise *t*-tests. ** *p < 0.01,* ***p < 0.001.

Figure 2 shows the muscle tone, stiffness, and PPT measurements for the two locations. According to the paired *t*-test, the TPs had significantly higher muscle tone and stiffness values compared with the control points (13.59 ± 1.37 Hz vs. 12.43 ± 1.21 Hz, *t* = 6.55, *p* < 0.001; 215.98 ± 40.25 N/m vs. 172.43 ± 30.18 N/m, *t* = 6.86, *p* < 0.001). The participants initially felt pain at a lower pressure at the TP compared with the control point (28.1 ± 9.15 N vs. 36.6 ± 11.04 N, *t* = −6.21, *p* < 0.001), indicating the hypersensitivity of the TP.

### 3.3. Psychophysical responses to deep compression at the TP and control point

**Figure 3:**
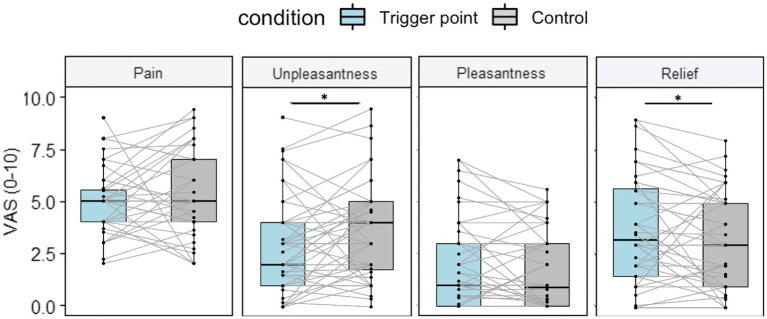
Psychophysical responses to deep compression at the TP and control point. The participants provided subjective ratings after deep compression at the TP and control point. Pain was not significantly different between the TP and control point (*p* = 0.51). Subjective relief was higher at the TP compared with the control point (*t* = 2.01, *p =* 0.05, 95% CI: 0 to 1.1). Pleasantness ratings were higher for the TP compared with the control point, although the effect size was small and the difference was not statistically significant (*d* = 0.239, *t* = 1.65, *p* = 0.1, 95% CI: −0.08 to 0.78). Unpleasantness ratings were significantly lower for the TP compared with the control point (*t* = −2, *p* = 0.01, 95% CI: −1.41 to −0.2). Boxplots show the median and interquartile range. All *p*-values were acquired via paired *t-*tests. * *p <* 0.05.

Figure 3 shows the subjective responses, i.e., VAS scores (0–10), to deep compression. As deep pressure was applied until the participants experienced moderate pain at each location, we expected to see similar pain levels between the TP and control point. There was no significant difference in pain between the TP and control point (5.01 ± 1.52 vs. 5.2 ± 2.01, *t* = −0.67, *p* = 0.51), which supported our hypothesis.

Deep compression at the TP was rated as significantly less unpleasant than that at the control point (2.78 ± 2.29 vs. 3.58 ± 2.26, *t* = −2.68, *p* < 0.05). In terms of subjective pleasantness, the participants rated TP stimulation as more pleasant than that at the control point, although the effect size was small (*d* = 0.24) and the results were not statistically significant (1.95 ± 2.07 vs. 1.6 ± 1.83, *t* = 1.65, *p* = 0.1). Participants experienced significantly more relief with TP stimulation compared with that at the control point (3.65 ± 2.62 vs. 3.1 ± 2.4, *t* = 2.01, *p* < 0.05).

### 3.4. Deep compression preference: TP versus control point

Of the 48 participants, 32 (66.7%) preferred compression at the TP over the control point, and the chi-square goodness of fit test revealed a significant difference in preference rates between the TP and control point (χ2 = 6.15, *p* = 0.01).

The participants were also asked whether they could distinguish between the TP and control points. Thirty-eight of the forty-eight participants (79.2%) indicated that they could not distinguish between the two points. Eight participants (16.8%) successfully identified the TP and two (4.2%) mistook the TP for the control point.

## 4. Discussion

This study investigated the properties of a TP and control point in the left brachioradialis muscle of healthy subjects. Muscle tone and stiffness, measured via myotonometry, had higher values for the TP compared with the control point. The PPT was significantly lower for the TP versus the control point, indicating that pain sensitivity was higher at the TP. The participants reported that unpleasantness was lower, and relief was higher, for compression at the TP compared with the control point, even though the perceived pain was not significantly different between the two areas.

The aim of this study was to investigate how deep compression of the TP and control point affects subjective perceptions of pain and pleasantness. Our results showed that deep compression at the TP was associated with lower unpleasantness ratings and more relief compared with that at the control point, even when the amount of perceived pain was the same. Previous studies have investigated affective responses to deep compression. A review on touch and massage therapy suggested that deep pressure can arouse positive feelings, such as pleasure, as well as brain activity similar to that observed during C-tactile touch (Field, 2019). A recent neuroimaging study investigated the pleasant experience of deep pressure application using an oscillating compression sleeve (Case et al., 2021). Deep pressure elicited similar affective ratings and brain activation to those of C-tactile touch. Morikawa et al. studied autonomic neural changes and prefrontal hemodynamic activity during deep compression in TPs and non-TPs (Morikawa et al., 2017). Subjective pain ratings were significantly lower in the TP compression condition compared with the non-TP compression condition, and the low frequency component of heart rate variability was decreased, while the high frequency component was increased, during TP compression. Also, brain activity data showed a significant decrease in prefrontal hemodynamic activity during TP compression compared with non-TP compression. The present study is the first to compare emotional responses to deep compression between a TP and control point, in addition to pain responses. Our results suggest that pain can be pleasant in certain circumstances, such as during the compression of TPs. While we also asked the participants at baseline about their massage preference, there was a high variability between participants, with a standard deviation of 24.21. In addition, no significant correlation was observed between the preference and the differences between the two points. Therefore, it is assumed that a person’s preference for massage may not be the cause of the differences in subjective ratings between TP and control. That is, in terms of the emotional reactions to deep compression, the location of the deep compression was more significant than individual differences in massage preference.

The participants in this study experienced pressure at the TP as more desirable than that at the control point. Specifically, they rated TP pressure as less unpleasant, indicated that it provided more relief, and gave it a higher overall preference rating compared with the control point, although the pleasantness rating was not significantly higher for the TP. This inverse relationship of preference ratings with those for unpleasantness and pleasantness may be seen for other treatments, such as acupuncture. Acupuncture stimulation usually causes pain rather than pleasant feelings. Nevertheless, some people benefit from acupuncture treatment (Bishop and Lewith, 2013). Neuroimaging studies of acupuncture have shown that responses to pain are modulated differently when pain is induced in a therapeutic context (Lee et al., 2015). Based on these studies, we suggest that some interventions may be preferred, and even pleasurable, even when the stimulus itself is not pleasant. However, the deep pressure in this study was given outside of a therapeutic context (the trials were conducted in an experimental setting) and the perceived pleasantness was low (the mean pleasantness score was <2 on a 10-point scale for both areas). Thus, further studies should conduct trials in more relaxing settings, such as the context of a therapeutic massage. In the current study, 79.2% of participants could not distinguish between TP and CT points. One possible reason is that this might be attributed by the closeness of location between two points. A previous study reported the two-point discrimination values of healthy participants in the posterior forearm as 30.7 ± 8.2 mm (Nolan, 1982). Therefore, we assume that the participant may find it challenging to identify TP or CT points at a distance of 2 cm.

This study is a pilot study conducted to explore the difference of emotional responses during similar level of pain in two points of the same muscle. We used deep compression, one of many interventions, because it works similarly to massage on TP. It is assumed that brain regions like the orbitofrontal cortex (OFC), anterior cingulate cortex (ACC), and hypothalamic regions may associate with the emotional responses during painful but less unpleasant touch. However, neuroimaging studies are required to explore the mechanism of this phenomenon. The OFC is known for reward and emotions like pleasure and can receive somatosensory input from the primary and secondary somatosensory cortex (Rolls et al., 2020). According to an fMRI study comparing the neurophysiological activity during pleasant touch, painful touch, and neutral touch, pleasant and painful touch activated the OFC more than neutral touch did, and these activations were linked to less activation in the primary somatosensory cortex. Another study on human touch massage compared 4 types of touch, including direct hand-to-hand contact, touch with or without movement and force, and touch with or without gloves. It found that direct hand-to-hand contact with movement had the highest level of pleasantness and activated the pregenual ACC (Lindgren et al., 2012). Studies on the effects of pressure massage on healthy volunteers have suggested that vagal activity and cortisol are both modulated by the hypothalamus (Ouchi et al., 2006). These findings are also consistent with a recent study that examined the impact of a novel treatment known as Musculoskeletal Inter-Fiber Counterirritant Stimulation, which aims to activate pain control pathways by repeatedly inducing ischemic compression, on participants with TP, general joint hypermobility, or joint hypermobility syndrome. All participants experienced analgesia from the treatment, which also modulated their autonomic nervous system by increased vagal activity and decreased sympathetic tone (Carvalho et al., 2022). In the current study, participants reported feeling less unpleasant and more relief in TP compared to control during the painful deep pressure compression. It is assumed that the desire for such pleasurable pain would be influenced by the reward mechanism and emotional processing from the OFC and ACC regions. Future research will be required to examine the neurophysiological mechanism of deep pressure stimulation on the TP in light of these findings.

TPs, with these psychophysiological and clinical characteristics, also encompass a range of physiological features. A previous study implementing ultrasound technology to distinguish the features of TPs to adjacent muscles found that TPs appear as one or more hypoechoic focal nodules on these images associated with blood flow disturbance (Sikdar et al., 2009). Sarcomeres in the middle of the TP region have been found to be short and constricted in histological analyses of TPs in both animal and human studies (Simons and Stolov, 1976; Jin et al., 2020). The precise mechanism underlying the development of TP is not yet fully understood. Research has hypothesized that long-term stress on the muscle can lead to sensitized nerve endings and dysfunctional endplates, resulting in sensitized loci on the muscle, while muscle contraction may restrict blood flow, which may potentially cause hypoxia in the affected area (Hong and Simons, 1998; Jafri, 2014). A recent study added to this hypothesis that muscle fatigue caused by overload or repetitive movement in the muscle increases the release of acetylcholine which can cause muscles to contract, or a rise in intracellular calcium levels, which can trigger the nociceptive response (Gerwin, 2023). Additionally, it is believed that TPs are associated with both peripheral and central sensitization. Referred pain is caused by the nociceptive signal from the peripheral to the dorsal horn neurons which can further initiate central sensitization (Xu et al., 2010; Fernandez-de-las-Penas and Dommerholt, 2014). In the future, it will be important to investigate the underlying anatomical properties and psychological traits of the TP area.

In the current study, an experienced Korean Medicine doctor precisely identify the locations of the TPs. A recent review discussed the lack of objective methods for quantifying lesions of the myofascial unit (Langevin, 2021). Multidisciplinary studies have focused on developing technologies to diagnose and predict biomarkers of myofascial pain. For instance, Reeves et al. used a pressure algometer to measure the sensitivity of myofascial TP (Albin et al., 2020), while Hong et al. evaluated the PPTs of latent TPs (LTrPs) and normal muscle tissue (Hong, 1998). They found that pressure pain threshold in LTrPs was lower compared with normal muscle tissue, and suggested that LTrPs could be detected via algometry (Hong, 1998). Recent studies introduced myotonometry, which is a non-invasive method for measuring the muscle response to a short mechanical stimulus (Pruyn et al., 2016; Jimenez-Sanchez et al., 2018). Another study focused on TPs on the infraspinatus in individuals with non-traumatic chronic shoulder pain, and reported higher muscle tone and stiffness at TPs compared with non-TPs; there was high intra- and inter-evaluator reliability, and good-to-excellent test–retest reliability (Roch et al., 2020). The results of the present study, i.e., lower PPT and higher muscle tone and stiffness in TPs, are in line with previous findings, indicating that the TPs were accurately detected. The diagnosis criteria used in this study was derived from a prior study that found that eliciting tenderness and the presence of a taut band are the most reliable TP features to identify during manual palpation, and can be the minimal criteria for identification (Gerwin et al., 1997). In order to visualize the area and location of TPs, it will be interesting to show a heatmap of the muscle properties, pain, and emotional responses in the target muscle in the future.

There were some limitations to this study. Because of the experimental setting, the therapeutic effects of deep compression may not have been fully realized. Consequently, we suggest that further studies examine the emotional responses to deep compression of TPs in a clinical setting. Also, it should be noted that this study is a pilot study investigating the emotional responses of deep pressure in trigger point. As this crossover study was conducted in one day, there are possibilities of carry-over effect from a previous condition. We attempted to minimize these effects by randomizing the order of TP and control for participants, and setting an interval between deep pressure of the two points. We examined all subjective ratings (pain, unpleasantness, pleasantness, and relief) and tested for order effects. We found no statistically significant differences between group A (TP first) and group B (control point first). These findings make it certain that participants might not be affected by a previous condition. To further compare the variations in responses during deep pressure in TPs and control points, however, we would need additional research in parallel group studies. Furthermore, because the participants were all healthy subjects, we cannot make inferences regarding the potential responses of subjects with myofascial lesions. Additional studies including subjects with musculoskeletal disorders may be required to assess the subjective responses to deep compression of TPs. Although all of our participants were healthy subjects, we nevertheless detected the LTrPs of the brachioradialis muscle. TPs include active TPs (ATrPs), which are more clinically significant because they produce spontaneous pain, and LTrPs, which are subclinical and cause pain only on stimulation (Celik and Mutlu, 2013). Although they are comparatively minor, LTrPs may progress to ATrPs, and the prevalence of LTrPs in healthy subjects is high (Hong and Simons, 1998; Lucas et al., 2010). Therefore, studies on LTrPs are meaningful. Finally, we only obtained subjective ratings of responses to deep compression. Future studies should examine the neurophysiological mechanisms underlying the response to deep compression of TPs.

In order for the researcher to maintain a steady pressure, we only used the force sensor to monitor a reliable, consistent pressure. However, force sensor test may be inaccurate when they are not performed on flat, rigid surfaces at room temperature (Schofield et al., 2016). As the sensor was placed on top of the participant’s forearm, the conditions of measurement differed between subjects and also between the two points of the same subject. Thus, we reasoned that the values were reliable only within the same condition of the same participant and used the force sensor to monitor the pressure and time during deep compression. However, in order to obtain more accurate pressure reports, we measured the pain threshold using a digital force gage. The pressure readings from the deep pressure compression were excluded from our main findings for these reasons.

This pilot study’s original objective was to investigate the phenomenon of pleasant pain in an experimental setting, more specifically when deep compression in TP was being applied. We noticed a phenomenon where people occasionally seek pressure out voluntarily, even when it hurts, like during a massage. The study procedure aimed to investigate this phenomenon by applying deep compression into TP and control, and recording the sensory and emotional responses during each trial. The fact that this study is the first to test the pleasant pain phenomenon in TP and deep compression makes it noteworthy even though this paper has some limitations, which will be further discussed in the discussion. A potential for pleasant pain during deep compression in TP has been discovered in preliminary research.

## 5. Conclusion

This study investigated the properties of TPs and control points in the brachioradialis muscle. Deep compression of TPs was rated as less unpleasant and higher pain relief. Our results imply that even if two stimulated sites are located in the same muscle, the emotional experience of pain can differ by its differences from myofascial properties. These findings advance our understanding of hedonic responses to pain.

## Data availability statement

The raw data supporting the conclusions of this article will be made available by the authors, without undue reservation.

## Ethics statement

The studies involving human participants were reviewed and approved by Institutional Review Board of Kyung Hee University. The patients/participants provided their written informed consent to participate in this study.

## Author contributions

SL, I-SL, and YC contributed to conception and design of the study. SL and HM performed the experiments. YR and I-SL conducted the statistical analysis. SL wrote the first draft of the manuscript. All authors contributed to the article and approved the submitted version.

## Funding

This research was supported by National Research Foundation of Korea (NRF) funded by the Ministry of Science, ICT & Future Planning (no. 2021R1F1A1046705) and Korea Institute of Oriental Medicine (KSN1812181) and by Institute of Information and Communications Technology Planning and Evaluation (IITP) grant funded by the Korea government (MSIT) [no. RS-2022-00155911, Artificial Intelligence Convergence Innovation Human Resources Development (Kyung Hee University)].

## Conflict of interest

The authors declare that the research was conducted in the absence of any commercial or financial relationships that could be construed as a potential conflict of interest.

## Publisher’s note

All claims expressed in this article are solely those of the authors and do not necessarily represent those of their affiliated organizations, or those of the publisher, the editors and the reviewers. Any product that may be evaluated in this article, or claim that may be made by its manufacturer, is not guaranteed or endorsed by the publisher.

## References

[ref1] Aggarwal A. Daniel J. Palekar T. J. (2020). Prevalence of myofascial trigger points in Brachioradialis, biceps Brachii, triceps Brachii, supinator and extensor carpi Radialis brevis in lateral epicondylitis. Indian J. Physiotherapy Occup. Therapy 14, 14–18. doi: 10.37506/ijpot.v14i1.3256

[ref2] Agyapong-Badu S. Aird L. Bailey L. Mooney K. Mullix J. Warner M.. (2013). Interrater reliability of muscle tone, stiffness and elasticity measurements of rectus femoris and biceps brachii in healthy young and older males. Working Papers Health Sci. 1, 1–11.

[ref3] Albin S. R. Koppenhaver S. L. MacDonald C. W. Capoccia S. Ngo D. Phippen S.. (2020). The effect of dry needling on gastrocnemius muscle stiffness and strength in participants with latent trigger points. J. Electromyogr. Kinesiol. 55:102479. doi: 10.1016/j.jelekin.2020.102479, PMID: 33075711

[ref4] Bailey L. Samuel D. Warner M. Stokes M. (2013). Parameters representing muscle tone, elasticity and stiffness of biceps Brachii in healthy older males: symmetry and within-session reliability using the MyotonPRO. Neurol. Dis. Ther. 1, 1–7. doi: 10.4172/2329-6895.1000116

[ref5] Bishop F. L. Lewith G. T. (2013). Patients' preconceptions of acupuncture: a qualitative study exploring the decisions patients make when seeking acupuncture. BMC Complement. Altern. Med. 13:102. doi: 10.1186/1472-6882-13-102 23664032 PMC3658911

[ref6] Cagnie B. Castelein B. Pollie F. Steelant L. Verhoeyen H. Cools A. (2015). Evidence for the use of ischemic compression and dry needling in the Management of Trigger Points of the upper trapezius in patients with neck pain: a systematic review. Am. J. Phys. Med. Rehabil. 94, 573–583. doi: 10.1097/PHM.0000000000000266, PMID: 25768071

[ref7] Cagnie B. Dewitte V. Coppieters I. Van Oosterwijck J. Cools A. Danneels L. (2013). Effect of ischemic compression on trigger points in the neck and shoulder muscles in office workers: a cohort study. J. Manip. Physiol. Ther. 36, 482–489. doi: 10.1016/j.jmpt.2013.07.001, PMID: 23993756

[ref8] Carvalho H. C. Machado N. Yanez-Silva A. Rocabado M. Junior A. R. P. Alves L. P.. (2022). Autonomic nerve regulation in joint hypermobility patients with myofascial trigger points by musculoskeletal Interfiber counterirritant stimulation (MICS). Med. Eng. Phys. 109:103903. doi: 10.1016/j.medengphy.2022.103903, PMID: 36371084

[ref9] Case L. K. Liljencrantz J. McCall M. V. Bradson M. Necaise A. Tubbs J.. (2021). Pleasant deep pressure: expanding the social touch hypothesis. Neuroscience 464, 3–11. doi: 10.1016/j.neuroscience.2020.07.050, PMID: 32768616 PMC7865002

[ref10] Celik D. Mutlu E. K. (2013). Clinical implication of latent myofascial trigger point. Curr. Pain Headache Rep. 17:353. doi: 10.1007/s11916-013-0353-8 23801006

[ref11] Cohen J. (1992). A power primer. Psychol. Bull. 112, 155–159. doi: 10.1037/0033-2909.112.1.155, PMID: 19565683

[ref12] Diego M. A. Field T. Sanders C. Hernandez-Reif M. (2004). Massage therapy of moderate and light pressure and vibrator effects on EEG and heart rate. Int. J. Neurosci. 114, 31–44. doi: 10.1080/00207450490249446 14660065

[ref13] Fernández-de-las-Peñas C. Alonso-Blanco C. Fernández-Carnero J. Page J. (2006). The immediate effect of ischemic compression technique and transverse friction massage on tenderness of active and latent myofascial trigger points: a pilot study. J. Bodyw. Mov. Ther. 10, 3–9. doi: 10.1016/j.jbmt.2005.05.003

[ref14] Fernández-de-las-Peñas C. Campo M. Fernández-Carnero J. Page J. (2005). Manual therapies in myofascial trigger point treatment: a systematic review. J. Bodyw. Mov. Ther. 9, 27–34. doi: 10.1016/j.jbmt.2003.11.001, PMID: 36498817

[ref15] Fernandez-de-las-Penas C. Dommerholt J. (2014). Myofascial trigger points: peripheral or central phenomenon? Curr. Rheumatol. Rep. 16:395. doi: 10.1007/s11926-013-0395-2 24264721

[ref16] Field T. (2014). Massage therapy research review. Complement. Ther. Clin. Pract. 20, 224–229. doi: 10.1016/j.ctcp.2014.07.002, PMID: 25172313 PMC5467308

[ref17] Field T. (2019). Social touch, CT touch and massage therapy: a narrative review. Dev. Rev. 51, 123–145. doi: 10.1016/j.dr.2019.01.002

[ref18] Gerwin R. D. (2023). A new unified theory of trigger point formation: failure of pre- and post-synaptic feedback control mechanisms. Int. J. Mol. Sci. 24:98142. doi: 10.3390/ijms24098142, PMID: 37175845 PMC10179372

[ref19] Gerwin R. D. Shannon S. Hong C. Z. Hubbard D. Gevirtz R. (1997). Interrater reliability in myofascial trigger point examination. Pain 69, 65–73. doi: 10.1016/s0304-3959(96)03248-4, PMID: 9060014

[ref20] Grabowski P. J. Slane L. C. Thelen D. G. Obermire T. Lee K. S. (2018). Evidence of generalized muscle stiffness in the presence of latent trigger points within infraspinatus. Arch. Phys. Med. Rehabil. 99, 2257–2262. doi: 10.1016/j.apmr.2018.03.024, PMID: 29709524

[ref21] Hong C. Z. (1998). Algometry in evaluation of trigger points and referred pain. J. Musculoskeletal Pain 6, 47–59. doi: 10.1300/J094v06n01_04

[ref22] Hong C. Z. Simons D. G. (1998). Pathophysiologic and electrophysiologic mechanisms of myofascial trigger points. Arch. Phys. Med. Rehabil. 79, 863–872. doi: 10.1016/S0003-9993(98)90371-9, PMID: 9685106

[ref23] Ibarra J. M. Ge H. Y. Wang C. Martinez Vizcaino V. Graven-Nielsen T. Arendt-Nielsen L. (2011). Latent myofascial trigger points are associated with an increased antagonistic muscle activity during agonist muscle contraction. J. Pain 12, 1282–1288. doi: 10.1016/j.jpain.2011.09.005, PMID: 22078789

[ref24] Jafri M. S. (2014). Mechanisms of myofascial pain. Int Sch Res Notices 2014:523924. doi: 10.1155/2014/523924, PMID: 25574501 PMC4285362

[ref25] Jimenez-Sanchez C. Ortiz-Lucas M. Bravo-Esteban E. Mayoral-Del Moral O. Herrero-Gallego P. Gomez-Soriano J. (2018). Myotonometry as a measure to detect myofascial trigger points: an inter-rater reliability study. Physiol. Meas. 39:115004. doi: 10.1088/1361-6579/aae9aa, PMID: 30475742

[ref26] Jin F. Guo Y. Wang Z. Badughaish A. Pan X. Zhang L.. (2020). The pathophysiological nature of sarcomeres in trigger points in patients with myofascial pain syndrome: a preliminary study. Eur. J. Pain 24, 1968–1978. doi: 10.1002/ejp.1647, PMID: 32841448 PMC7693045

[ref27] Kao M. J. Han T. I. Kuan T. S. Hsieh Y. L. Su B. H. Hong C. Z. (2007). Myofascial trigger points in early life. Arch. Phys. Med. Rehabil. 88, 251–254. doi: 10.1016/j.apmr.2006.11.004, PMID: 17270525

[ref28] Langevin H. M. (2021). Fascia mobility, proprioception, and myofascial pain. Life (Basel) 11:70668. doi: 10.3390/life11070668, PMID: 34357040 PMC8304470

[ref29] Lavelle E. D. Lavelle W. Smith H. S. (2007). Myofascial trigger points. Anesthesiol. Clin. 25, 841–851, vii-iii. doi: 10.1016/j.anclin.2007.07.003, PMID: 18054148

[ref30] Lee I. S. Wallraven C. Kong J. Chang D. S. Lee H. Park H. J.. (2015). When pain is not only pain: inserting needles into the body evokes distinct reward-related brain responses in the context of a treatment. Physiol. Behav. 140, 148–155. doi: 10.1016/j.physbeh.2014.12.030, PMID: 25528104

[ref31] Leknes S. Berna C. Lee M. C. Snyder G. D. Biele G. Tracey I. (2013). The importance of context: when relative relief renders pain pleasant. Pain 154, 402–410. doi: 10.1016/j.pain.2012.11.018, PMID: 23352758 PMC3590449

[ref32] Lidbeck J. (2002). Central hyperexcitability in chronic musculoskeletal pain: a conceptual breakthrough with multiple clinical implications. Pain Res. Manag. 7, 81–92. doi: 10.1155/2002/310974, PMID: 12185372

[ref33] Lindgren L. Westling G. Brulin C. Lehtipalo S. Andersson M. Nyberg L. (2012). Pleasant human touch is represented in pregenual anterior cingulate cortex. NeuroImage 59, 3427–3432. doi: 10.1016/j.neuroimage.2011.11.013, PMID: 22100768

[ref34] Lucas K. R. Rich P. A. Polus B. I. (2010). Muscle activation patterns in the scapular positioning muscles during loaded scapular plane elevation: the effects of latent myofascial trigger points. Clin. Biomech. (Bristol, Avon) 25, 765–770. doi: 10.1016/j.clinbiomech.2010.05.006, PMID: 20667633

[ref35] Mackenzie N. (2009). A phenomenological study of women who presented to a physiotherapy led continence service with dyspareunia and were treated with trigger point massage. J. Assoc. Chartered Physiotherapists Women's Health 105, 24–39.

[ref36] Moraska A. F. Hickner R. C. Kohrt W. M. Brewer A. (2013). Changes in blood flow and cellular metabolism at a myofascial trigger point with trigger point release (ischemic compression): a proof-of-principle pilot study. Arch. Phys. Med. Rehabil. 94, 196–200. doi: 10.1016/j.apmr.2012.08.216, PMID: 22975226 PMC3529849

[ref37] Moraska A. F. Schmiege S. J. Mann J. D. Butryn N. Krutsch J. P. (2017). Responsiveness of myofascial trigger points to single and multiple trigger point release massages: a randomized, placebo controlled trial. Am. J. Phys. Med. Rehabil. 96, 639–645. doi: 10.1097/PHM.0000000000000728, PMID: 28248690 PMC5561477

[ref38] Morikawa Y. Takamoto K. Nishimaru H. Taguchi T. Urakawa S. Sakai S.. (2017). Compression at myofascial trigger point on chronic neck pain provides pain relief through the prefrontal cortex and autonomic nervous system: a pilot study. Front. Neurosci. 11:186. doi: 10.3389/fnins.2017.00186, PMID: 28442987 PMC5386976

[ref39] Nolan M. F. (1982). Two-point discrimination assessment in the upper limb in young adult men and women. Phys. Ther. 62, 965–969. doi: 10.1093/ptj/62.7.965, PMID: 7089059

[ref40] Ouchi Y. Kanno T. Okada H. Yoshikawa E. Shinke T. Nagasawa S.. (2006). Changes in cerebral blood flow under the prone condition with and without massage. Neurosci. Lett. 407:131-135. doi: 10.1016/j.neulet.2006.08.037 16973270

[ref41] Pawling R. Cannon P. R. McGlone F. P. Walker S. C. (2017). C-tactile afferent stimulating touch carries a positive affective value. PLoS One 12:e0173457. doi: 10.1371/journal.pone.0173457, PMID: 28282451 PMC5345811

[ref42] Pruyn E. C. Watsford M. L. Murphy A. J. (2016). Validity and reliability of three methods of stiffness assessment. J. Sport Health Sci. 5, 476–483. doi: 10.1016/j.jshs.2015.12.001, PMID: 30356566 PMC6188913

[ref43] Raja S. N. Carr D. B. Cohen M. Finnerup N. B. Flor H. Gibson S.. (2020). The revised International Association for the Study of Pain definition of pain: concepts, challenges, and compromises. Pain 161, 1976–1982. doi: 10.1097/j.pain.0000000000001939, PMID: 32694387 PMC7680716

[ref44] Roch M. Morin M. Gaudreault N. (2020). The MyotonPRO: a reliable tool for quantifying the viscoelastic properties of a trigger point on the infraspinatus in non-traumatic chronic shoulder pain. J. Bodyw. Mov. Ther. 24, 379–385. doi: 10.1016/j.jbmt.2020.05.002, PMID: 33218538

[ref45] Rolls E. T. Cheng W. Feng J. (2020). The orbitofrontal cortex: reward, emotion and depression. Brain Commun 2:fcaa196. doi: 10.1093/braincomms/fcaa196 33364600 PMC7749795

[ref46] Schneider S. Peipsi A. Stokes M. Knicker A. Abeln V. (2015). Feasibility of monitoring muscle health in microgravity environments using Myoton technology. Med. Biol. Eng. Comput. 53, 57–66. doi: 10.1007/s11517-014-1211-5, PMID: 25331739

[ref47] Schofield J. S. Evans K. R. Hebert J. S. Marasco P. D. Carey J. P. (2016). The effect of biomechanical variables on force sensitive resistor error: implications for calibration and improved accuracy. J. Biomech. 49, 786–792. doi: 10.1016/j.jbiomech.2016.01.022, PMID: 26903413 PMC5903557

[ref49] Sikdar S. Shah J. P. Gebreab T. Yen R. H. Gilliams E. Danoff J.. (2009). Novel applications of ultrasound technology to visualize and characterize myofascial trigger points and surrounding soft tissue. Arch. Phys. Med. Rehabil. 90, 1829–1838. doi: 10.1016/j.apmr.2009.04.015, PMID: 19887205 PMC2774893

[ref50] Simons D. G. Stolov W. C. (1976). Microscopic features and transient contraction of palpable bands in canine muscle. Am. J. Phys. Med. 55, 65–88. PMID: 1266956

[ref51] Suresh S. Wang S. Porfyris S. Kamasinski-Sol R. Steinhorn D. M. (2008). Massage therapy in outpatient pediatric chronic pain patients: do they facilitate significant reductions in levels of distress, pain, tension, discomfort, and mood alterations? Paediatr. Anaesth. 18, 884–887. doi: 10.1111/j.1460-9592.2008.02638.x 18768049

[ref52] Suvilehto J. T. Glerean E. Dunbar R. I. Hari R. Nummenmaa L. (2015). Topography of social touching depends on emotional bonds between humans. Proc. Natl. Acad. Sci. U. S. A. 112, 13811–13816. doi: 10.1073/pnas.1519231112, PMID: 26504228 PMC4653180

[ref53] Takakura N. Takayama M. Kawase A. Kaptchuk T. J. Yajima H. (2013). Double-blind acupuncture needle: a potential tool to investigate the nature of pain and pleasure. ISRN Pain 2013:825751. doi: 10.1155/2013/825751, PMID: 24288658 PMC3839571

[ref54] Vieira A. I. Ramos A. V. Cavalheiro L. M. Almeida P. Nogueira D. Reis E.. (2016). Reliability and validity of the European Portuguese version of the social touch questionnaire. J. Nonverbal Behav. 40, 363–377. doi: 10.1007/s10919-016-0239-7, PMID: 27818562 PMC5075018

[ref55] Xu Y. M. Ge H. Y. Arendt-Nielsen L. (2010). Sustained nociceptive mechanical stimulation of latent myofascial trigger point induces central sensitization in healthy subjects. J. Pain 11, 1348–1355. doi: 10.1016/j.jpain.2010.03.010, PMID: 20451466

