# Supplementary material for: Sensory and emotional responses to deep pressure stimulation at myofascial trigger points: a pilot study
Source: Front Neurosci. 2023 Jul 7;17:1197302. doi: 10.3389/fnins.2023.1197302 (PMC10360180; doi:10.3389/fnins.2023.1197302)

**Supplementary Figure 1**

**The spatial patterns of muscle stiffness.**

The Myoton PRO device (Myoton AS, Tallinn, Estonia) was used to evaluate muscle stiffness over the region of interest (Myofascial trigger points: TP and Control points) over the brachioradialis muscle. The area of interest measures 5 × 5 cm. The value of muscle stiffness was visualized using Matlab R2020a software (MathWorks, Natick, MA, USA). Data on muscular stiffness suggest that a 2 cm spacing between the control point (green dot) and the target point (TP: red dot) may be sufficient. One participant provided the measurement of muscular stiffness. This heatmap can only be displayed to help with understanding the localizing TP and control points. Grey dotted-line represented brachioradialis muscle.


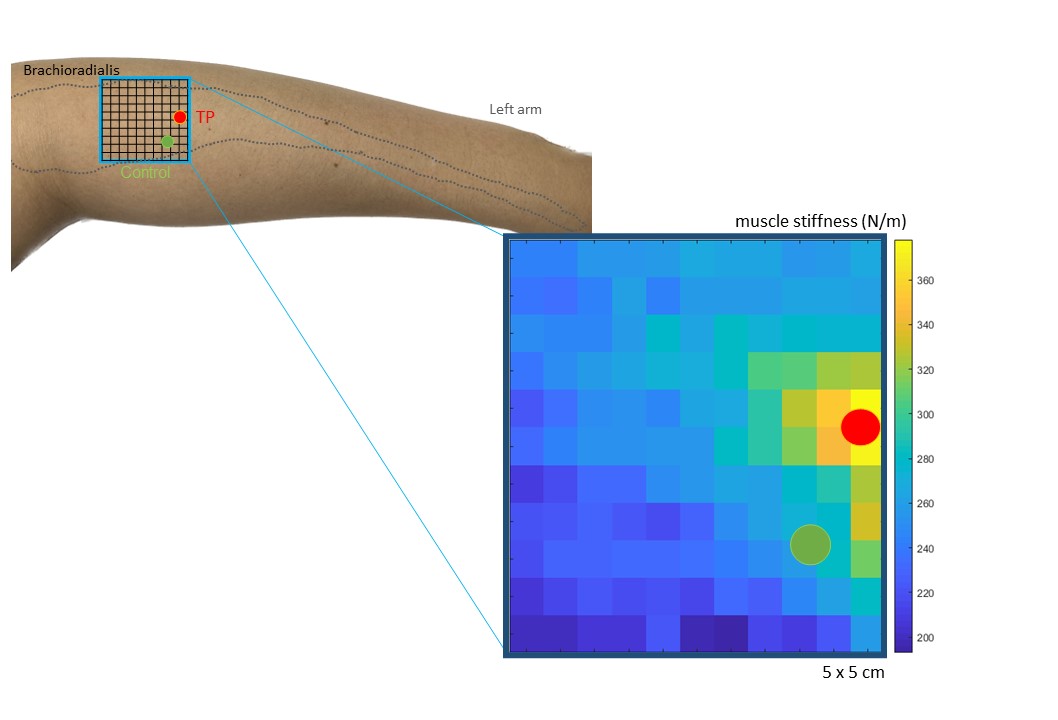

Supplement: Supplementary file 1 [file Table_2.DOC]
